# Surface plastic flow in polishing of rough surfaces

**DOI:** 10.1038/s41598-019-46997-w

**Published:** 2019-07-23

**Authors:** Ashif S. Iquebal, Dinakar Sagapuram, Satish T. S. Bukkapatnam

**Affiliations:** Texas A&M University, Department of Industrial & Systems Engineering, College Station, 77843 Texas, USA

**Keywords:** Mechanical engineering, Surfaces, interfaces and thin films

## Abstract

We present experimental evidence for a new mechanism for how smooth surfaces emerge during repetitive sliding contacts, as in polishing. Electron microscopy observations of Ti-6Al-4V surface with a spherical asperity structure—realized via additive manufacturing—during successive polishing stages suggest that asperity-abrasive contacts exhibit viscous behavior, where the asperity material flows in the form of thin (1–10 *μ*m) fluid-like layers. Subsequent bridging of these layers among neighboring asperities results in progressive surface smoothening. Using analytical asperity-abrasive contact temperature modeling and microstructural characterization, we show that the sliding contacts encounter flash temperatures of the order of 700–900 K which is in the range of the dynamic recrystallization temperature of the material considered, thus supporting the experimental observations. Besides providing a new perspective on the long-held mechanism of polishing, our observations provide a novel approach based on graph theory to quantitatively characterize the evolution of surface morphology. Results suggest that the graph representation offers a more efficient measure to characterize the surface morphology emerging at various stages of polishing. The research findings and observations are of broad relevance to the understanding of plastic flow behavior of sliding contacts ubiquitous in materials processing, tribology, and natural geological processes as well as present unique opportunities to tailor the microstructures by controlling the thermomechanics of the asperity-abrasive contacts.

## Introduction

Mechanical interactions between severely rubbing surfaces have long been of fundamental interest for understanding friction in a wide range of domains including tribology, materials processing and geophysics. An important practical application of such interactions is in polishing of materials where rubbing action of fine abrasives is utilized to obtain smooth surfaces for application in optics, microscopy and mechanical instrumentation.

The practice of polishing to impart solid surfaces with smooth, lustrous finish has been known for centuries. The use of hard abrasives such as corundum and diamond for polishing in fact dates back to the Neolithic period^1^ and Leonardo da Vinci is credited with the earliest systematic design of a polishing machine^2^. It might be surprising then to know that the mechanism of polishing—how surface irregularities are smoothened out by abrasive particles—is still unsettled. Excellent account of the history and theories of polishing can be found in^3–5^. However, it may suffice to note that mainly two lines of thought for the polishing mechanism have prevailed: that of abrasion and surface flow. Early theories by Hooke and Newton^6^, followed by those of Herschel^5^ and Rayleigh^7^ viewed polishing essentially as an abrasion or a grinding process at a very fine scale where surface irregularities are removed by cutting action of the abrasives. The work by Samuels^8^ presented irrefutable evidence for this mechanism and showed how abrasives act as planing tools and result in the generation of well-defined chips as they slide past a surface. However, the fine scale abrasion theory falls short in explaining the high (almost an order of magnitude) compressive residual stresses obtained after polishing as compared to milling and finish machining operations^9^.

The alternative theory emerges from the work by Beilby^10^ who proposed surface smoothening occurring via surface flow and material redistribution. Here, it is believed that the material from surface peaks ‘flows’ to fill up the valleys and forms a thin vitreous surface layer, generally referred to as the “Beilby layer”. Bowden and Hughes^11^ further developed this theory and proposed that surface flow is in fact mediated by local melting at the surface–abrasive contacts. Electron diffraction measurements of polished surfaces have been presented as indirect evidence for the Beilby layer formation, but these observations were later proved to be inconclusive. To our knowledge, no conclusive evidence for the surface flow or melting has been provided to date. Other theories of polishing also exist, among which noteworthy is the molecular level material removal mechanism put forward by Rabinowicz^4^ based on energy considerations.

More recently, the emergence of additive manufacturing (AM) technology has renewed the interest in polishing processes^12,13^. AM technologies are severely limited in terms of creating controlled surface morphology^14^ and suffers from poor surface quality and porosity issues^15^. Consequently, the existing AM technologies are typically coupled with some form of post-processing mechanism to improve the surface finish and reduce surface porosity. Mechanical polishing is one of the most commonly employed post-processing approaches in this context. Surface finish requirements vary depending upon the applications, from biomedical implants that call for differential surface roughness (sub-micron finish at bearing locations while rough, textured surface at the bone-implant interaction site^16^) to mechanical applications that require a uniform specular finish (average surface roughness, *S*_*a*_ < 25 nm). Therefore, it is important to understand the exact phenomenology governing surface modification during the post-processing stages and develop accurate modeling approaches to achieve the desired specifications.

However, unlike machining processes such as milling and turning, modeling and simulation of the polishing process is extremely challenging due to the complexities arising from the stochastic nature of the asperity-abrasive interaction (such as depth of cut and rake angle) as well as the deformation mode at asperities (abrasion versus surface material flow). Additionally, in the context of AM surfaces, where surface roughness is almost two orders of magnitude higher, little to no studies exist on the mechanism of surface modification during polishing. Lack of a unified theory hinders accurate modeling of the surface modification during polishing. For example, if polishing is driven purely via abrasion, it is highly unlikely to get rid of surface pores as the subsurface pores (created during the metal powder sintering process) would get exposed on continued material removal. On the contrary, if surface material redistribution is considered as the driving mechanism for surface smoothening, then opportunities may exist to exploit surface plastic flow in polishing processes to minimize residual surface porosity in AM components.

In this study, we present direct experimental evidence that support the surface plastic flow as the dominant mechanism in the polishing of rough metal surfaces, fabricated via an AM process (electron beam melting, EBM). We present scanning electron microscopy (SEM) observations of polished surfaces that reveal viscous flow at the asperity-abrasive sliding contacts, involving material flow towards the asperity sides in the form of thin fluid-like layers. The subsequent stages of polishing involve bridging of these layers among different asperities to result in a smooth finish. The observations suggest that as polishing ensues, surface smoothening is mediated mostly via material redistribution as opposed to material removal. We establish that the viscous flow at the asperity-abrasive contact is mediated by the high flash temperatures of the order of 700–900 K which is in the range of dynamic recrystallization temperature of the material considered, thus supporting the experimental observations. To determine the flash temperatures generated from frictional heating during the sliding of carefully tuned (spherical) asperity surface against the abrasives we use a circular moving heat source model. By taking into account the distribution of abrasive and asperity profiles, we show that such high flash temperatures are highly likely (>33%) to occur during the repetitive interactions. We also conduct *ex post facto* microstructural analysis to further support the occurrence of viscous flow at the asperity-abrasive interface and present possible microscopic mechanisms underlying the plastic flow during the polishing process.

In the sequel, we also show that these observations form the basis for a pseudo-physics-based model to quantitatively capture the complex evolution of surface morphology during polishing using random planar graphs. We show that this graph-theoretic quantification and the corresponding spectral characteristics correctly capture the progression of the bridging process and smoothening of surfaces during polishing. Results show that the spectral characteristics of the resulting graphs serve as an efficient indicator of the process endpoints as compared to the standard surface roughness measures used in the industry. We also shed some light on the possibility of viscous flow as a general mechanism in other sliding contacts.

## Results

### Surface plastic flow

**Figure 1:**
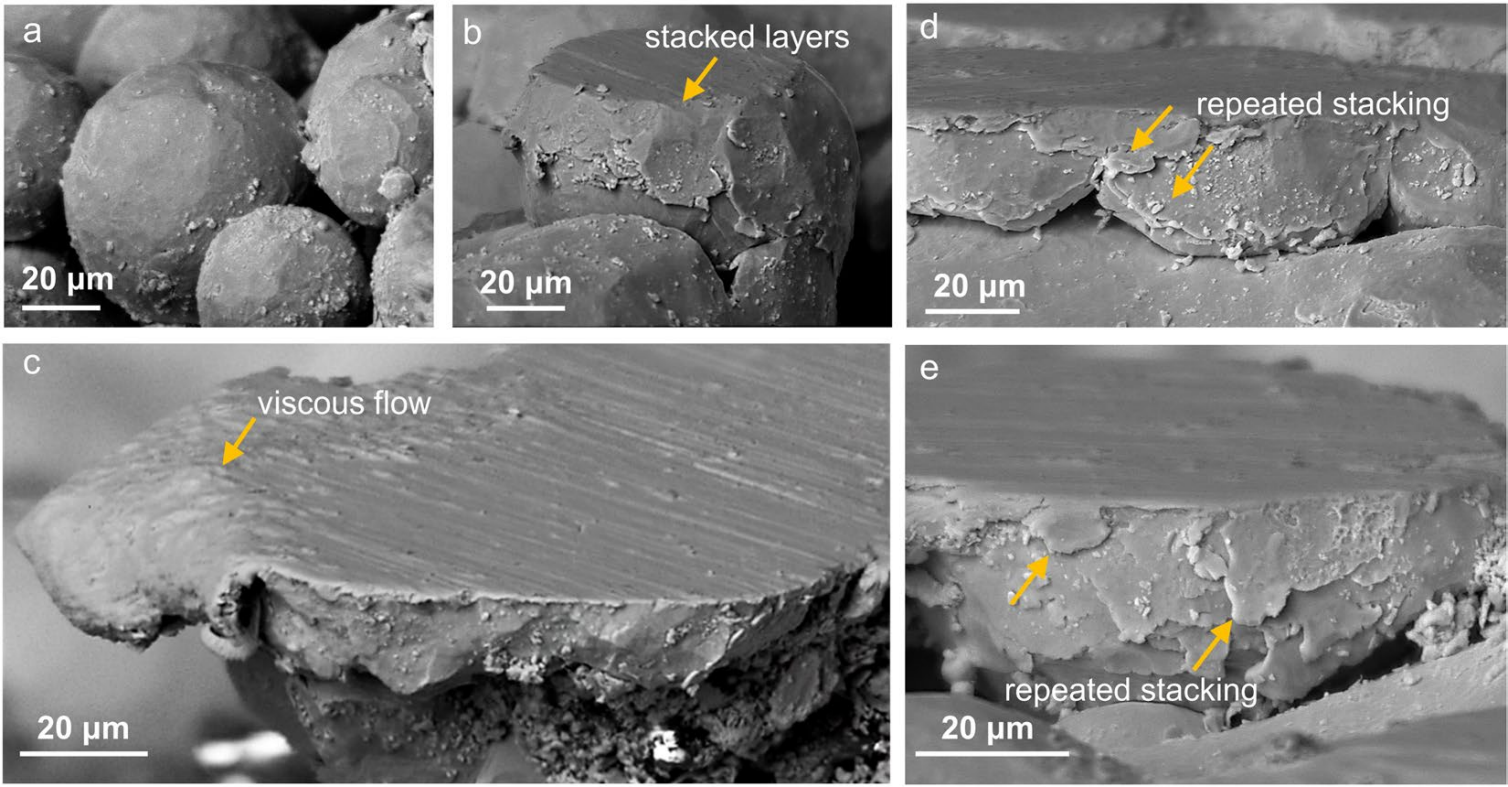
SEM images showing (**a**) the spherical asperity structure at *t* = 0 s, (**b**,**c**) surface asperities after 90 s of polishing showing the evidence of complex material flow patterns: (**b**) lateral plastic flow and deposition of material on the asperity sides, (**c**) shear deformation of the asperity surface and material flow towards the edge of the asperity in the form of thin, fluid-like layers of the order of 1–10 *μ*m and (**d**,**e**) surface asperities showing repeated formation and deposition of thin material layers as the asperity progressively flattens out on continued polishing.

Electron microscopy of the surface asperities enabled us to capture key phenomenological details of the polishing process. Based on these observations, we subdivide the polishing process into four different stages as discussed in the following. These stages may exist at the same time depending upon the characteristics of asperity-abrasive interaction, e.g., contact area, asperity height, etc. Polishing begins with the interaction between the abrasive particles and the asperities, initially spherical as shown in Fig. 1(a). At this stage, material removal could be observed macroscopically, however, the electron microscopic investigation shows that in addition to the material removal, thin layers of material begin to stack on the asperity sides. Figure 1(b,c) show typical asperity structures after 90 s of polishing. Severe shear of the asperity surface and accumulation of the material towards asperity edges (see at arrow) is evident from Fig. 1(c). This flow pattern is reminiscent of plastic sliding between surfaces oriented at shallow angles, such as in tribological contacts or ‘machining’ under highly negative rake angles^17,18^. The sheared surface material then flows to the lateral sides of the asperity as thin layers, usually in the range of 1–10 *μ*m. Interestingly, the flow is seen to be quite symmetric around the periphery of the sheared surface, with deposited material layer showing a molten-like appearance. The sliding direction between the asperity and abrasive particle can be inferred from the sliding marks in Fig. 1(c). This omnidirectional flow at the surface, coupled with the observation of rheological flow features at the asperity edges (Fig. 1(c)), suggests viscous behavior of the surface plastic flow in polishing.

The electron microscopic investigation of the subsequent polishing stages provides further evidence of the surface flow theory in that the surface smoothening is mediated by material redistribution more so than material removal. Figure 1(d,e) show the progression of the plastic flow at the asperity surface on continued polishing (beyond 90 s). This forms the second stage of the polishing process. The repeated shearing at the asperity surface upon encountering a sliding abrasive result in the stacking of multiple thin layers on the lateral sides of the asperity (see at arrow). In effect, this lateral flow of material results in a radial increase in the flattened area of the asperity.

**Figure 2:**
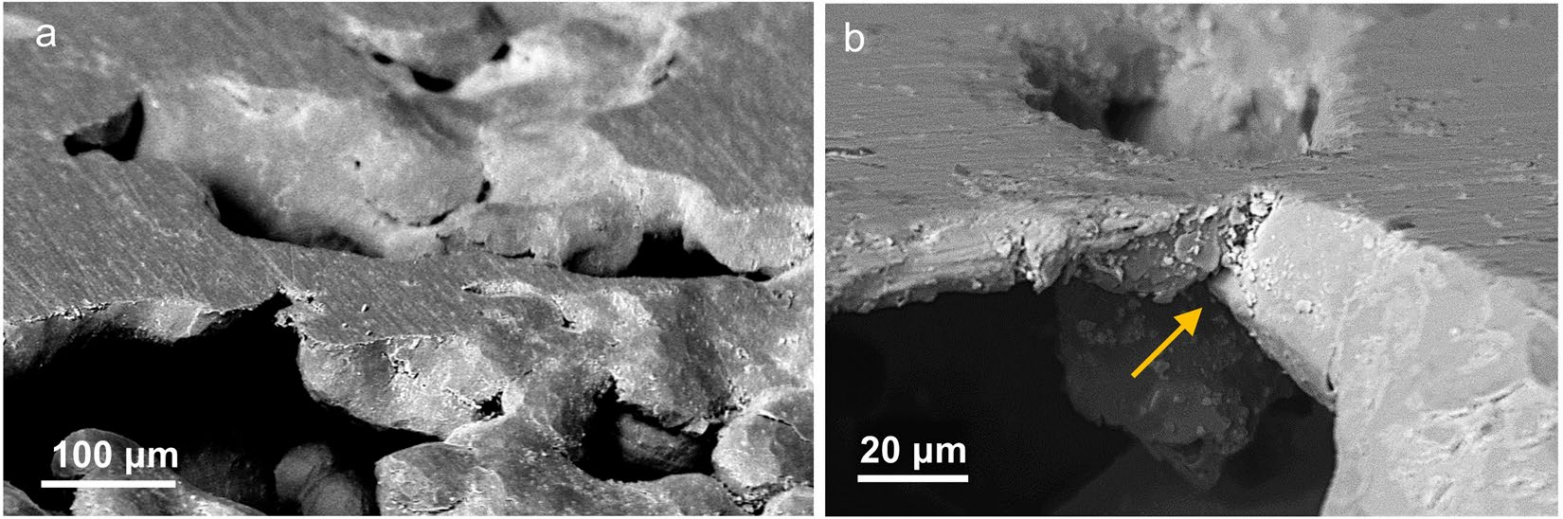
Surface smoothening during later stages of polishing by bridging between neighboring asperities: (**a**) SEM image showing the interconnection of flat (“smooth”) regions surrounded by unfilled depressions; (**b**) a high-magnification image of the bridge (see at arrow) that has formed between neighboring asperities.

**Figure 3:**
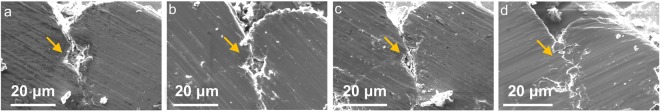
SEM observations of the temporal evolution of a representative surface depression ~10 *μ*m in size (indicated by arrow). Images in (**a**–**d**) are taken repeatedly at the same location at 90 s time interval beginning with *t* = 180 s). The depression is progressively filled up as a result of material flow from the neighboring surface.

Figure 2 illustrates the surface morphology characteristics on continued polishing, at 180 s. In this third stage of polishing, individual asperity surfaces are unresolvable, and the surface can be described as an interconnected network of flat islands as seen in Fig. 2(a). Interspersed among these regions are the unfilled depressions. A closer inspection of the flattened regions (Fig. 2(b)) reveals that their formation is mediated by bridging of the smeared surface material between the neighboring asperities. Therefore, the third stage of the polishing process is characterized by the bridging of neighboring asperities. Indeed, this “welding” between the asperities may be expected given the occurrence of severe plastic flow and temperatures (see Section 3.2) at the asperity surfaces. In our experiments, this bridging phenomenon was noted only when the distance between the edges of two neighboring asperities approached 30 *μ*m. For asperities separated by larger distances, lateral flow of the material was seen to continue until the effective distance between the asperities approached the critical value. Continued polishing causes complete bridging of individual asperities, resulting in a nominally smooth surface (for example, see Fig. 6-top row). This final stage (fourth stage) in the polishing involves elimination of microscale depressions. Flattening of these microscopic depressions during the final stage of polishing again seems to occur as a result of material flow from neighboring flat regions. A series of SEM images showing the progression of a representative surface depression is presented in Fig. 3. The images were taken at the same location repeatedly at 90 s time interval beginning with *t* = 180 s. We note that the effective diameter of the depression gradually decreases as a result of the material flow from the neighboring flat surface, that is, surface depressions are smoothened out via plastic flow of the neighboring surface and not by abrasion. This is also in agreement with the observations presented in^19^ where the authors showed, in a similar fashion, closing of a microindentation mark after repeated sliding contacts.

The observations presented in the foregoing suggest that polishing primarily involves material redistribution in the form of thin fluid-like layers towards the asperity sides as well as during the bridging process. In contrast, material removal (or abrasion) is mostly limited to the initial stages of polishing.

### Asperity-abrasive contact temperature

**Figure 4:**
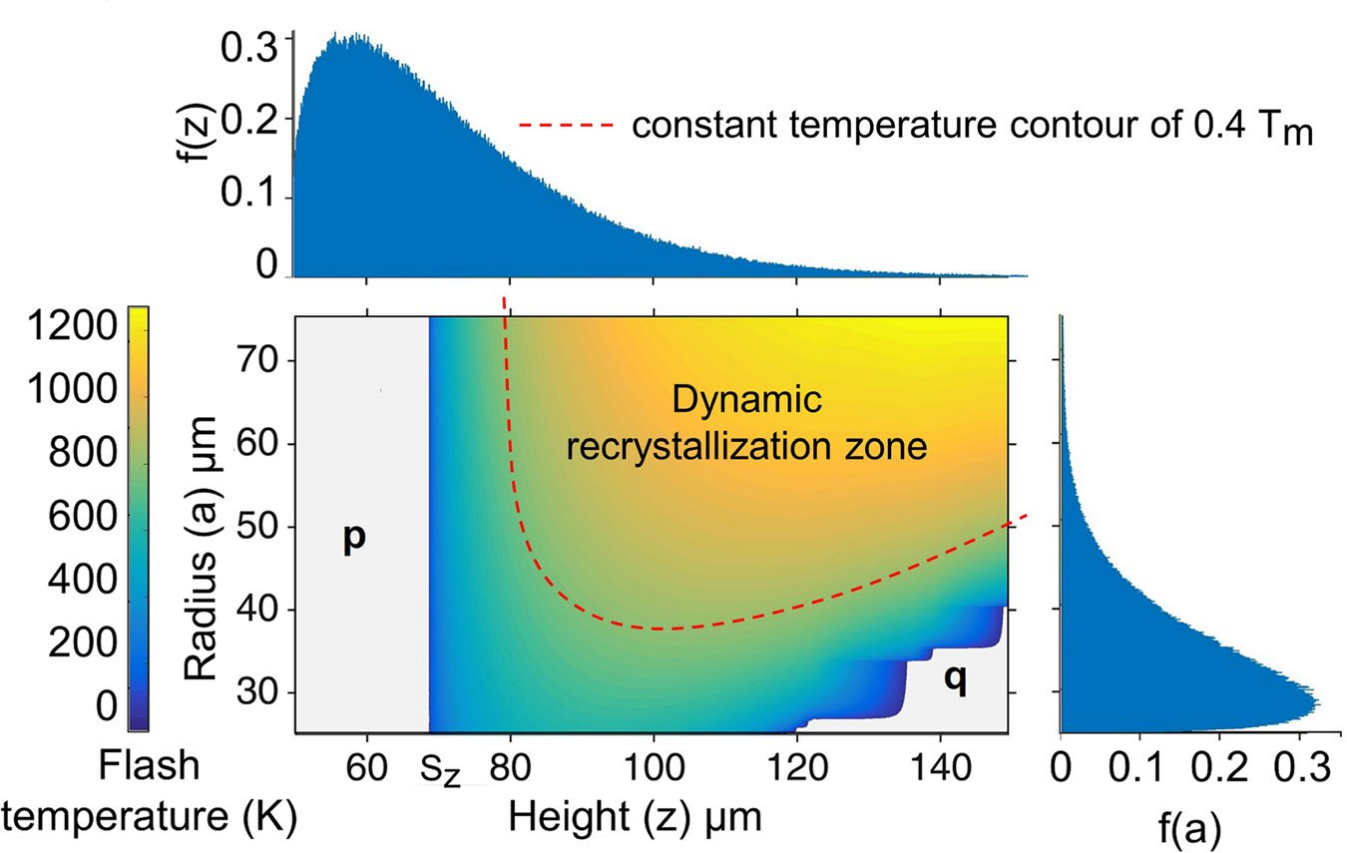
Flash temperature map for Ti-6Al-4V as a function of asperity-abrasive contact radius (*a*) and height (*z*), both of which follow a truncated Weibull distribution with average at 36 *μ*m and 64.5 *μ*m, respectively, and a standard deviation ~15 *μ*m. *S*_*z*_ corresponds to the average asperity height. The dynamic recrystallization zone (≥0.4*T*_*m*_) is also indicated on the temperature map.

To explore the possible origin for this flow behavior, we estimated the “flash” temperature at the asperity-abrasive sliding contacts using the circular moving heat source model^20^, where the abrasive particle was treated as a semi-infinite moving body over which a stationary heat source acts. The heat source intensity was taken as the heat dissipation due to plastic shearing of the asperity at the sliding asperity-abrasive contact. See Supplementary Information (section S1) for details on the flash temperature calculations. The flash temperature map as a function of the asperity height *z* and the asperity-abrasive contact radius *a* is shown in Fig. 4. Any asperity not involved in the polishing process either makes no contact with the abrasive or lie outside the asperity-abrasive contact region. These regions are marked as “**p**” and “**q**”. Elsewhere, we notice that larger values of *a* and *z* result in higher flash temperatures.

From Fig. 4, we note that the calculated flash temperatures for ~30% of the sliding contacts were above 700 K. While these temperatures are well below the melting temperature (*T*_*m*_ =  1925 K) of Ti-6Al-4V, they are in the typical dynamic recrystallization temperature range (700–900 K) for this alloy where significant flow softening occurs^21^. At such temperatures, rate-dependent viscous plastic flow is not uncommon in metals^22^. Recent studies^23^ show that the flow softening is primarily caused due to the formation of dynamically recrystallized nanograins with very low dislocation density within the shear bands. Similar fluid-like flow phenomenon in metals have been also noted previously in other sliding configurations^24,25^ and shear bands^26–28^.

### Microstructural analysis

**Figure 5:**
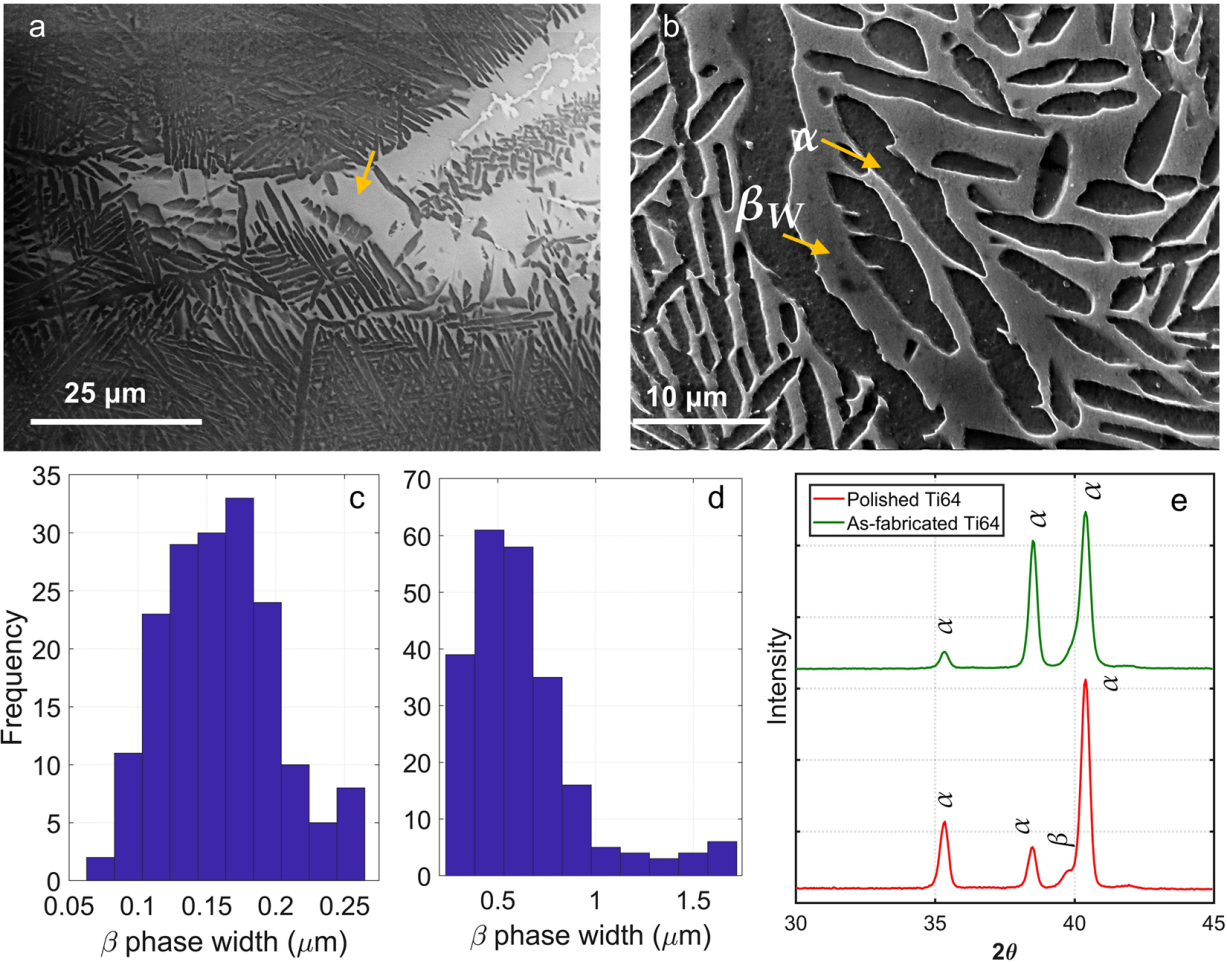
Microstructural characterization of the polished surface. (**a**,**b**) SEM images showing extreme coarsening at scattered locations (see arrow in (**a**)) and overall widening of the *β* phase (≥3 *μ*m average width). (**c**) and (**d**) show the width distribution of the *β* phase before and after polishing, respectively. (**e**) XRD profile of the surface in as-fabricated (top, green) and polished (bottom, red) condition.

SEM analysis of the microstructure further supports the predictions that flash temperatures above 700 K are possible. In comparison to the as-fabricated microstructure (see Fig. 8(d)), we observe regions with significant coarsening of the *β* phase on the polished surface (Fig. 5(a)). *β* phase with an average width of 0.7 *μ*m was observed along with a significant decrease in the volume fraction of *α* phase from ~90% before polishing to ~60% after polishing. This is also evident from the distribution of the widths of *β* phase before and after polishing as shown in Fig. 5(c,d). This transformation is likely due to the high flash temperatures in the range of 700–90 K during polishing, coupled with extremely high cooling rates (~10^6^ K/s) as estimated from the moving heat source model. Additionally, such high flash temperatures can cause migration of *β* colony boundaries resulting in coarsening of the *β* phase as noted in Fig. 5(a). Furthermore, polished surfaces are also often characterized by regions of very coarse *β* phase, a fact that is consistent with the stochastic nature of asperity-abrasive interactions where flash temperatures may last from less than 10 *μ*s to several seconds (due to repeated rubbing)^29^.

Widening of the *β* phase is further confirmed by the X-ray diffraction of the polished surface as shown in Fig. 5(e). Higher concentration of *β* phase is evident in the XRD profile of the polished surface as compared to the as-built sample. It has also been shown in^30^ that the emergence of *β* phase in XRD profile was observed when the sample was heated in the range of ~1200 K. The microstructure evolution observed in the polished samples suggests that the work hardening (as a consequence of dislocation generation) during polishing is likely to be accompanied by some dislocation annihilation mechanism, akin to annealing at high temperatures. Given the flash temperatures at the asperity-abrasive contacts are in the range of ~0.4*T*_*m*_, dynamic recrystallization/recovery may be the dominant mechanism in this regard. An important consequence of repeated surface plastic flow is the refinement of *α* lamellae at the surface and the associated increase in the strength. Indeed, hardness measurements (Vickers indentation, load 500 g) showed the surface to be characterized by a higher hardness (375 kg/mm^2^) compared to the base material (350 kg/mm^2^).

### Microscopic view of the plastic flow

Plastic deformation in metals and alloys generally takes place via an interplay between generation, motion, and annihilation of dislocations^31^. Given the stochastic nature of the polishing process, many of these deformation mechanisms may co-exist. In this section, we utilize our estimates of temperature coupled with the order-of-magnitude analysis of typical strain rates and stresses to identify the dominant mechanisms underlying the plastic flow during the polishing process.

Given the surface plastic strains^8^ in polishing are well above 1, at a polishing speed of 5 m/s, the strain rate ($\dot{\gamma }$) should be of the order of 10^3^–10^5^ s^−1^. At such high strain rates and typical flash temperatures of about 900 K, the dislocations motion should be drag controlled, with the drag on the mobile dislocations primarily arising from phonon interactions^32^. For example, in phonon-limited dislocation glide the strain rate is proportional to *ρ*_*m*_*σ*_*s*_/*B* where *σ*_*s*_ is the applied stress, *ρ*_*m*_ is the mobile dislocation density (≈10^15^–10^17^ m^−2^ for heavily deformed metals) and *B* is the phonon viscosity drag coefficient (≈10^−5^ Pa⋅s)^33^. Considering an average applied stress of 100 MPa, the estimated strain rate under the phonon limited glide is about 10^4^ s^−1^, which is within the expected range of strain rate. A similar analysis for diffusion-based mechanisms (e.g., lattice or grain boundary diffusion) reveals that these mechanisms are likely to be minor contributors to the flow at strain rates and temperatures relevant to polishing. However, at flash temperatures exceeding 1200 K, dislocation motion via climb as well as grain boundary sliding (GBS) are the plausible secondary mechanisms that could contribute to the plastic flow. Recent studies have shown that GBS can also accommodate some other deformation modes, including grain rotation^34^ and twinning^35^. Delineation of the individual contributions of these various mechanisms to the overall plastic flow in polishing is a challenging task that remains to be tested.

### Random planar graph representation of surface morphology

In this section, we show how the SEM observations of the surface flow and material redistribution presented in the foregoing lends itself to a random planar graph representation of the evolution of surface morphology. More specifically, the merger of neighboring asperities via the bridging process offers a natural possibility of a graph-based representation where asperities can be represented as nodes and the likelihood of asperities to merge represents edge weights connecting these nodes. Such a representation is important from a surface quality assurance and process characterization standpoint to enable endpoint detection and prediction capabilities in the finishing of additively manufactured components^12^.

**Figure 6:**
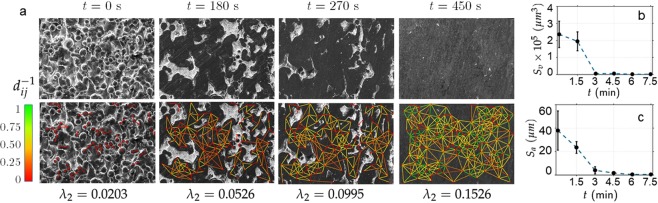
Evolution of Surface morphology during polishing. (**a**) SEM images (top row) and corresponding network representation (bottom row) showing the gradual asperity bridging process. The corresponding Fiedler value, *λ*_2_ (which quantifies the extent of connectivity among the asperities) is also given. The colormap represents the inter-asperity distance (*d*_*ij*_ = ||*v*_*i*_ − *v*_*j*_||_2_) as measured from the asperity periphery, and shows bridging of farther asperities during the later stages of polishing. The plots in (**b**,**c**) show the temporal evolution of the volume of the voids (*S*_*v*_) and the surface roughness (*S*_*a*_) during polishing.

Figure 6(a, top row) summarizes the surface morphological evolution during the entire duration of polishing. The micrographs show the surface flow and bridging among the asperities, together with the gradual reduction of the volume of inter-asperity valleys (light regions). Bridging results in a strongly inter-connected network of flat areas (dark regions) that eventually evolve to form a uniformly smooth surface (with average roughness, *S*_*a*_~30 nm). In essence, the phenomena of bridging of the neighboring asperities represent an evolving random planar graph *G*(*t*) = (*V*, *E*(*t*)) where the nodes *V* denote the asperities and the edge weights *E*(*t*) denote the propensity of a pair of neighboring nodes to bridge, evolving over time. Details on the calculation of *E*(*t*) may be found in the Supplementary Information (section S2).

From the *in situ* SEM image sequence shown in Fig. 6(a), we notice that as the polishing process ensues, asperities progressively bridge and is reflected by an increase in the connectivity of the representative asperity network (Fig. 6(b)). To quantify this evolution pattern (we show in the sequel that this quantification would also serve to validate the planar random graph model), we track the second smallest eigenvalue *λ*_2_ (also called the Fiedler value^36^) of the graph Laplacian, $L(t)\triangleq {\mathscr{D}}(t)-E(t)$ where ${\mathscr{D}}(t)$ is the diagonal matrix representing the degree of each node and is given as:

1 $${\mathscr{D}}(t)=[\begin{array}{llll}\sum _{j=1}^{N}\,{e}_{1j}(t) & \sum _{j=1}^{N}\,{e}_{2j}(t) & \ldots  & \sum _{j=1}^{N}\,{e}_{Nj}(t)\end{array}]$$

where *e*_*ij*_(*t*) are the elements of the matrix *E*(*t*) and *N* is the total number of nodes. Fiedler value serves as a natural quantifier to capture the evolution in the neighborhood structure and consequently the effects of polishing on the surface morphology, particularly during the bridging process. For example, *λ*_2_ = 0 indicates the complete absence of bridge formation; in contrast, *λ*_2_ ≥ 0.15 suggests a higher degree of bridging where every node is connected to at least six other neighboring nodes representing a close packing of spherical asperities. Readers may refer to^12^ for detailed information on the relationship between *λ*_2_ and the node degree. The micrograph patterns as well as the corresponding *λ*_2_ values presented in Fig. 6(a) suggest that as polishing ensues and the asperity diameters grow, the propensity of neighboring asperities to bridge (i.e., *e*_*ij*_(*t*)) progressively increases. Quantitatively speaking, the initial value of *λ*_2_ = 0.0203 (see Fig. 6(a), bottom row) indicates little bridging (average number of bridges connecting a node or the “degree” is <1) as reflected in *e*_*ij*_(*t*) being close to zero between almost all asperities. Specifically, the edges connecting the neighboring nodes are almost absent initially, and low probability edges (red) connect only a sparse set of neighboring nodes. After 450 s of polishing, *λ*_2_ increases to 0.1526, suggesting a higher degree of bridging among all neighboring asperities (degree ≥6), and high *e*_*ij*_(*t*) values.

The corresponding temporal evolution of volume of inter-asperity “valleys” *S*_*v*_ and average surface roughness *S*_*a*_, captured using surface interferometry, are given in Fig. 6(b,c), respectively. While both *S*_*v*_ and *S*_*a*_ decrease monotonically with time, *S*_*v*_ drops sharply from ~2 × 10^5^ *μ*m^3^ to 2.1 × 10^3^ *μ*m^3^ between 90 s and 180 s (Fig. 6(b)). This corresponds to the time interval where bridging of the asperities is predominant (see Fig. 2). Unlike *S*_*v*_, the value of *S*_*a*_ continues to decrease even after 180 s, likely because of surface smoothening via reduction in microscale surface depressions during the final stages of polishing (see Fig. 3).

## Discussion

The foregoing observations of surface morphology suggest that material redistribution is the dominant mechanism of polishing as opposed to material removal, especially for extremely rough surfaces that are peculiar to conventionally adopted additive manufacturing processes (layer by layer deposition of metal powder). Analytical investigations of asperity-abrasive flash temperatures as well as the microstructural evolution (widening of the *β* phase) suggest that the formation of thin fluid-like viscous layers is driven by the occurrence of high flash temperatures in the range of 700–900 K.

### Mechanism of polishing and generality of the observations

It may be noted that our observations of surface plastic flow and material redistribution presented in this work are somewhat contrary to the conventional theories of polishing, originally advocated by Hooke, Newton^6^, Herschel^5^ and Rayleigh^7^, viewing polishing essentially as an abrasion or a grinding process at a very fine scale. Here, surface irregularities were believed to be removed by cutting action of the abrasives. Studies presented by Samuels and Aghan^8,37^ showed that polishing and abrasion are phenomenologically the same process and differed only in the degree of material removal. Direct observations of the polished copper surfaces under SEM demonstrated the formation of micro-chips that established cutting and ploughing as the dominant mechanisms of surface smoothening^38^. The study also established that the molecular material removal theory proposed by Rabinowicz^4^ is highly unlikely to occur.

Interestingly, however, our observations point to an alternate surface flow and material redistribution theory of polishing proposed by Beilby and Bowden^10^. However, several important distinctions are noted with respect to this theory. First, no evidence for surface melting or amorphization was noted in contrast to the original hypotheses^10,11^, although the microscopy observations of the surface flow profiles, together with the temperature calculations of the asperity-abrasive sliding contacts, strongly suggest the occurrence of viscous flow. Second, as demonstrated in Fig. 1, the material redistribution is facilitated by the material flow as thin layers (1–10 *μ*m) that make self-contact with the asperity sides. This is again at variance with the original ideas where the surface valleys are believed to be filled purely via compression (and lateral flow) of the asperities. Lastly, bridging among asperities is seen to be an important mechanism by which neighboring asperities merge to form a smooth surface network.

We note that the material properties, as well as the starting asperity structure obtained via AM (that was not feasible in the previous studies), may play a key role in determining the dominant mechanism in polishing. For example, the low thermal diffusivity of Ti-6Al-4V (≈3.6 × 10^−6^ m^2^/s) undoubtedly contributes to the high temperatures localized near the asperity-abrasive interface, causing sufficient softening and material flow. We believe that it is the lack of controlled starting asperity structure (to track unit plastic flow events) and the limitations on the microscopic power that contributed to the dismissal of Beilby’s surface flow theory^8^. While other factors such as down force and the polishing speed could also influence the findings presented in the paper, e.g., flash temperature rise and the subsequent viscous flow, the values chosen for polishing load and speed in our study are representative of most metallographic polishing processes^8^. Among others, such as lubricants only control the nominal surface temperature rise and has been shown to have negligible effect on the local flash temperatures^39^.

**Figure 7:**
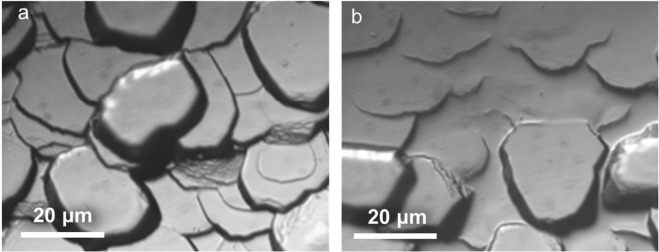
Scanning electron micrographs showing surface morphological changes in Ta_2_O_5_: (**a**) before and (**b**) after polishing.

Nonetheless, we believe that the current findings are likely to be more generic to the polishing of a range of other material systems. This is supported by the fact that surface flow profiles at the asperities similar to that in Figs 1 and 2 were also observed in polishing an entirely different material system: tantalum oxide (Ta _2_O _5_), see Fig. 7. These observations in oxide materials, while at first surprising given their inherent brittle behavior, can be explained by the high asperity-abrasive contact pressures that typically exceed the work piece material’s hardness. It is well known that such high pressures can, in turn, promote plastic flow even in highly brittle materials^40^. Additionally, the asperity-abrasive contact temperature calculations for polishing of Ta_2_O_5_ showed that the flash temperatures can be a significant fraction (~0.4*T*_*m*_) of its melting temperature, which could potentially enhance the propensity for viscous-type flow at asperity surfaces. Nonetheless, understanding the mechanism behind such flows require further studies and still remains an open problem. Besides polishing, our observations are also of relevance to a range of other engineering and physical systems where microscale asperity contacts, characterized by high pressures, are of intrinsic interest, e.g., tribological systems, erosion, and earthquakes.

### Quantifying evolution of surface morphology

Application of analytical approaches to quantify the material structure and mechanical properties offer exciting avenues for optimal material and process design^14,41^. In this direction, the random planar graph approach presented in the foregoing allows quantification of surface morphology at different polishing stages and a robust route to endpoint detection. Most of the existing analytical models for surface quality estimation are based on material removal theories and therefore are inadequate in characterizing the surface morphology at the sub-micron level. Conventional quantifiers such as *S*_*a*_ do not capture the evolution of micropores and voids, especially towards the end stages of polishing. For the experiments considered in this work, the *S*_*a*_ values showed an almost negligible change of 0.37% in contrast to 3.2% change in *λ*_2_ value during the last 90 seconds of the polishing process. From an endpoint detection standpoint, this suggests that *S*_*a*_ may not be a consistent and accurate representation of surface quality improvements. In contrast, we note that the *λ*_2_ value is more sensitive to the neighborhood structure, i.e., the distance of neighboring asperities (presence of porosity results in a non-zero distance), and consequently the *λ*_2_ value would increase as long as the surface roughness and porosity reduces.

## Conclusions

The physics by which rough surfaces are smoothened during polishing has remained a controversial topic because of the inherent complexity and the stochastic nature of the process. The speculations on the exact phenomenology of the polishing process, therefore, have largely been based on the postmortem analysis of the polished surfaces. However, no conclusive evidence exists till now to support either the surface flow or the fine scale abrasion (material removal) theory.

In this work, we have presented experimental characterization, thermal and microstructural studies to settle this paradox by gathering direct evidence of basic flow events through which surface morphological changes occur in polishing. Using a carefully designed (spherical) asperity structure, we analyze the deformation state of the asperities after every 90 s of the polishing process using scanning electron microscopy. Towards this end, we present observations that not only provide conclusive evidence for the general Beilby–Bowden’s surface flow picture, but also bring out new phenomenology of polishing pertaining to viscous flow at asperity-abrasive contacts, self-contact of *flown* layers with the asperity sides, and subsequent bridging among these layers that closely resembles evolution of a complex network. Subsequently, the stochastic circular moving heat source model as well as the microstructural observations suggest that the formation of viscous thin-fluid like layers is driven by the occurrence of high flash temperatures of the order of 700–900 K that are in the range of dynamic recrystallization temperature of the material system (Ti-6Al-4V) considered in this study. These results altogether establish an alternative mechanism of surface smoothening that is mediated by viscous flow and redistribution of surface asperities and contradicts the widely adapted fine scale abrasion and chip formation theory.

The experimental observations have also allowed us to quantitatively capture the evolution of surface morphology during the polishing process using a random planar graph theoretic approach. Results suggest that the spectral characteristic (*λ*_2_) of the planar graph is a better quantifier of the surface morphology and an efficient estimator of the process endpoint as compared to the average surface roughness, particularly in the case of rough metal surfaces. These results are significant as accurate predictions of process endpoints and surface morphological modeling are becoming crucial in the additive manufacturing industry where post-processing efforts account for over 25% increase in the production costs and 17–100% increase in the cycle times^12^.

The new perspective on surface plastic flow and bridging phenomena presented in this work creates opportunities to exploit the viscous flow behavior of polishing to tailor the mechanical properties along with controlling the surface roughness and porosity. From an analytical perspective, these observations can also help in developing models that can accurately predict the surface morphologies at different polishing stages by accounting for material redistribution rate along with the material removal.

## Materials and Methods

### Surface preparation

**Figure 8:**
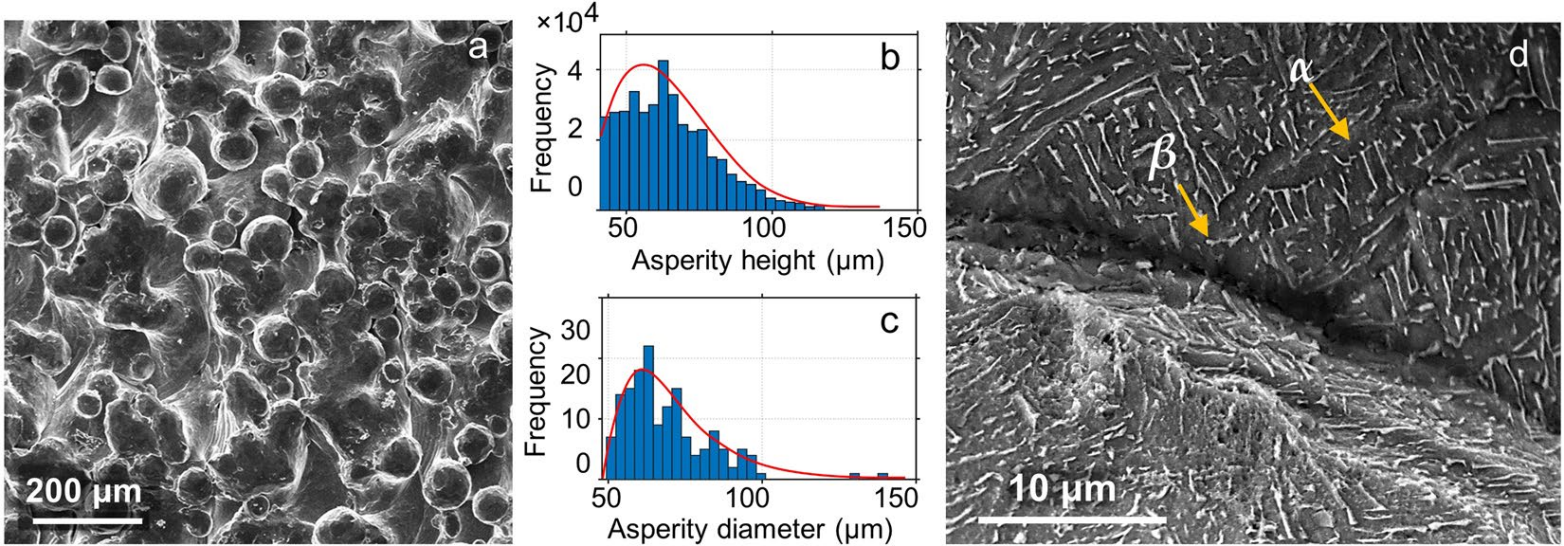
Surface morphology characteristics of Ti-6Al-4V alloy sample prepared using the EBM process. (**a**) SEM image showing the spherical asperity structure of the sample surface; (**b**,**c**) are respectively the distribution plots for the asperity height and diameter as measured using white light interferometry. Empirical estimate suggests a Weibull distribution with an average value of 72 *μ*m and 64.5 *μ*m, respectively and a standard deviation of ~15 *μ*m. (**d**) SEM image showing the representative *α* + *β* colony with *α* lamellae (in dark) interspersed with *β* phase (rod-shaped, in white) of average thickness 200 nm.

Ti-6Al-4V samples of 50 mm and 7 mm thickness with controlled surface morphology consisting of spherical asperity structure were prepared using an Arcam EBM machine operating at a vacuum of 2 Pa and accelerating voltage of 60 kV. The process involved raking a 50 *μ*m layer of Ti-6Al-4V powder of average 72 *μ*m (see Fig. 8(c) for the distribution of diameter of Ti-6Al-4V particles) using a focused electron beam of 3 mA, scanning at a speed of 10 m/s. The resulting surface consists of granular Ti-6Al-4V particles with a unique spherical asperity structure. SEM image of the representative surface morphology is shown in Fig. 8(a). The distributions of asperity height and diameter are shown in Fig. 8(b,c), respectively. The asperity height as well as the diameter exhibit a Weibull distribution with an average value of 72 *μ*m and 64.5 *μ*m, respectively, and a standard deviation of ~15 *μ*m. Incidentally, the idealization of surfaces as a collection of spherical asperities (with Gaussian and Weibull distribution of heights) has been the basis for many prior theoretical analyses of elastic-plastic contacts between rough surfaces^42^.

### Surface smoothening via polishing

The Ti-6Al-4V samples were polished on a Buehler Metaserv Grinder-Polisher (model 95-C2348-160) using silicon carbide (SiC) polishing pads (203 mm), in stages, with progressively smaller abrasives ranging from 30 *μ*m to 5 *μ*m under dry conditions. A steady nominal down pressure of 0.5 kPa was maintained and the polisher speed was fixed at 500 rpm. The surface was manually subjected to a quasi-random orbital motion. The final polishing step involved the use of alumina abrasives (<1 *μ*m), suspended in an aqueous solution (20% by wt., pH ≈7.5) for 20 minutes to impart a specular finish to the surface. The slurry was routinely flushed to avoid possible agglomeration of finer abrasives into large clusters that may result in scratching.

### Surface and microstructure characterization

The polishing was interrupted at every 90 s intervals to observe the surface morphology changes and the evolution of asperity structure using a Zeiss EVO scanning electron microscope. Quantitative details pertaining to the surface finish including surface roughness (*S*_*a*_) and volume of inter-asperity “valleys” (*S*_*v*_) were measured using a white light interferometer from Zegage. Inter-asperity valleys were characterized by the surface heights lying below the 10^th^ percentile on the bearing area curve (i.e., the cumulative distribution of surface profile)^42^. To ensure that observations and measurements were made at the same surface location during different polishing steps, the sample surface was initially indented with a 2 × 2 mm square grid. The vertices of this grid enabled us to image the same surface location after each interrupted test. To facilitate better observations of the plastic flow patterns at asperity surfaces, the sample was tilted by 70° in the scanning electron microscope.

The hardness of the polished sample was measured on a Leco LM300 AT microhardness machine with a load and dwell time of 500 g and 5 s, respectively. To observe the microstructures, the mechanically polished samples were first treated with Kroll’s reagent (5–7% nitric acid (HNO_3_) and 2–4% hydrofluoric acid (HF) and rest distilled water) for 10 s and then rinsed with distilled water. The microstructures were observed using SEM under high vacuum (≤1 × 10^−4^ mbar) with accelerating voltage of 15 kV and a working distance of 15 mm. Figure 8(d) shows the representative microstructure observed in an as-EBM fabricated Ti-6Al-4V, comprising of lamellar *α* and *β* phases with both colony and basket-weave (also called Widmanstätten pattern) morphology^43^. The *α* lamellar phase is etched out by Kroll’s reagent and therefore, exhibits a darker contrast as compared to the *β* phase under SEM. Here, the *β* phase resembles rod-like morphology with an average thickness of 200 nm.

## Supplementary information

Supplemental material

## Data Availability

All the data used in this work are available upon request.
