# Supplementary material for: Surface plastic flow in polishing of rough surfaces
Source: Sci Rep. 2019 Jul 23;9:10617. doi: 10.1038/s41598-019-46997-w (PMC6650475; doi:10.1038/s41598-019-46997-w)
Supplement: Supplementary file 1 — Supplemental material [file 41598_2019_46997_MOESM1_ESM.pdf]

Supplemental material: Surface plastic flow in polishing of rough surfaces

Ashif S. Iquebal*, Dinakar Sagapuram, and Satish Bukkapatnam

Department of Industrial and Systems Engineering,

Texas A&M University, College Station, Texas 77840, USA

ashif_22@tamu.edu, dinakar@tamu.edu, satish@tamu.edu

S1: Calculation of flash temperatures at the asperity–abrasive contacts

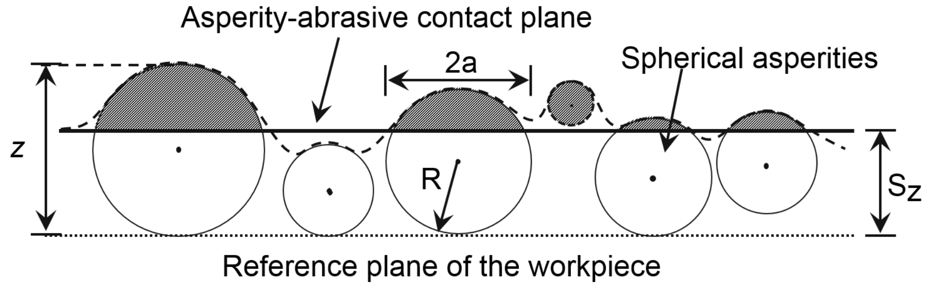

FIG. S1. Schematic showing contact (solid line) between the workpiece surface consisting of spherical asperities and the polishing pad at a distance S_z (average asperity heights) from the workpiece reference plane (dotted line). Here, the asperity height, z, is measured with respect to the workpiece reference plane.

For a given asperity height (z) distribution, only the asperities for which $z > S_z$ and $z \leq S_z + 2R$ are involved in the polishing process, as schematically shown in Fig. S1. Here, the asperity height, z, is measured with respect to the workpiece reference plane (dotted line

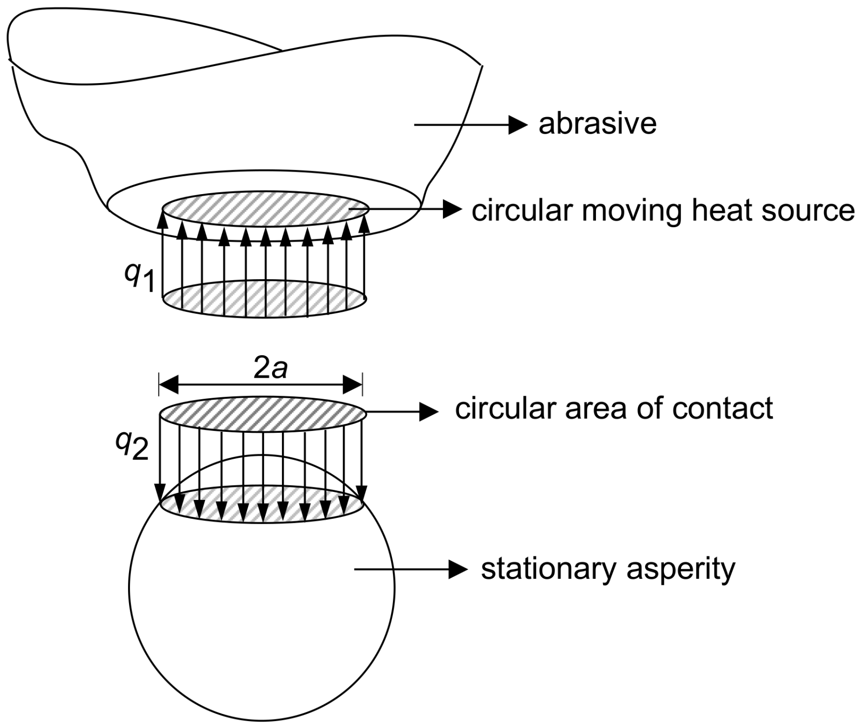

FIG. S2. Moving circular heat source model for the contact between asperity and abrasive to calculate the temperature rise during polishing. Here, the abrasive is considered as the semi-infinite moving heat source and the asperity acts as a stationary heat source.

in the schematic in Fig. S1). We assume that the clearance between the workpiece reference plane and the polishing pad (solid line) is equal to the average surface asperity heights, S_z, of the workpiece. The diameter of asperity–abrasive contact ($2a$) can then be calculated for a given value of S_z, asperity radius (R) and height (z) distribution.

Given the radius of contact, we calculate flash temperature by treating the contact as a moving circular heat source (Fig. S2). The heat source intensity is taken as the heat dissipation due to plastic shearing of the metal asperity at the sliding interface. The heat partition between the asperity and the abrasive particle is determined by setting equal the maximum (quasi-steady state) temperatures of the asperity and abrasive particle within the contact, according to Blok's postulate [S1]. Here, we treat the abrasive as a semi-infinite moving body (with velocity V) over which a stationary heat source (with uniform heat flux) acts. The steady state flash temperature occurring at the contact center can accordingly be given by the first order approximation to Jaegar's circular moving heat source model [S2, S3] as:

$$\Delta T_{max}\big|_{abrasive} = \frac{2q_2 a}{k_2\sqrt{(\pi(P_{e2}+1.273))}} \tag{1}$$

where, Peclet number, $P_{e2} = Va/2K_2$ and $K_2 = k_2/\rho_2 C_2 \approx 4 \times 10^{-5}$ m^2/s. For $V = 5$ m/s and contact radius a, we have $P_{e2} = 6.25 \times 10^5 a$. For the asperity (which is treated as a stationary source), we have:

$$\Delta T_{max}\big|_{asperity} = \frac{q_1 a}{k_1} \tag{2}$$

Assuming adiabatic conditions, where plastic dissipation at the interface is completely converted into heat, the total heat flux, q, at the circular contact is given by:

$$q = q_1 + q_2 = \mu HV \tag{3}$$

By equating the maximum temperatures at the asperity and abrasive surface, we have:

$$\Delta T_{max} = \frac{\mu HVa}{k_1}\left(1 + \frac{k_2}{2k_1}\sqrt{\pi(P_{e2}+1.273)}\right)^{-1} \tag{4}$$

We solve for ΔT_{max} for Ti-6Al-4V using the values in Table 1, and the corresponding flash temperature map as a function of asperity height and abrasive-asperity contact radius

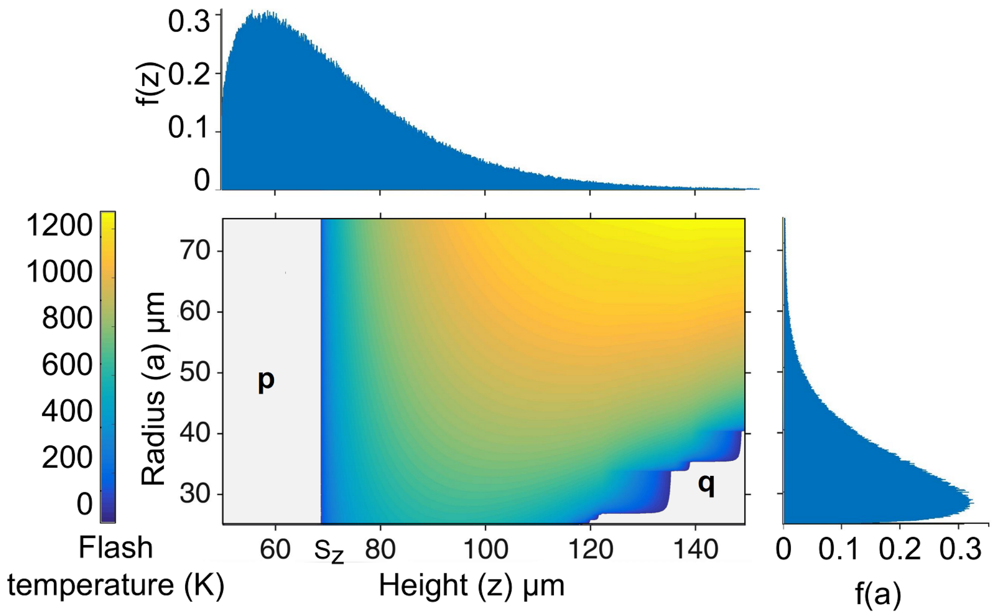

FIG. S3. (a) Flash temperature map for Ti-6Al-4V as a function of abrasive-asperity contact radius and height, both of which follow a truncated Weibull distribution with average at 36 μm and 64.5 μm, respectively, and a standard deviation $\sim$ 15 μm. S_z corresponds to the average asperity height.

is shown in Fig. S3(a). Any asperity for which $z < S_z$ or $z \geq S_z + 2R$ would not be involved in the polishing process as it would either make no contact with the abrasive or lie outside the asperity–abrasive contact region (solid line in Fig. S1). These two cases are marked as "**p**" and "**q**" in Fig. S3(a). Elsewhere, we notice that larger values of R and z result in higher flash temperatures.

While the assumption of abrasive as a semi-infinite plane maybe reasonable during the initial stages of polishing, the configuration is reversed as polishing process progresses. During the intermediate and final stages, polishing maybe represented as individual abrasive particles sliding across a semi-infinite workpiece surface. For this latter configuration, we assume abrasive particles as sliding conical indenters plastically deforming the workpiece surface. Again for this case, the problem is that of a moving semi-infinite body (workpiece surface) over which stationary heat source (abrasive-workpiece surface contact) acts. The maximum flash temperature rise at the contact in this case is given as:

$$\Delta T_{max} = \frac{\mu H V a}{k_2}\left(1 + \frac{k_1}{2k_2}\sqrt{\pi(P_{e1} + 1.273)}\right)^{-1} \quad (5)$$

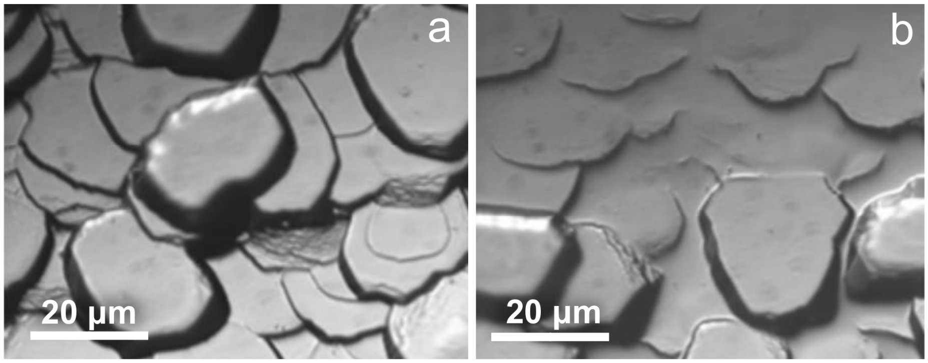

FIG. S4. Scanning electron micrographs showing surface morphological changes in Ta_2O_5: (a) before and (b) after polishing.

The calculated sliding temperatures for this configuration are slightly larger than those in the earlier configuration where abrasive was taken as a semi-infinite plane (Fig. S2). The difference between temperature estimates for these two configurations is within 20% (at a contact radius of $\sim$40 μm) for the contact areas considered here. In both the configurations, for $\sim$ 30% of the sliding contacts, maximum flash temperatures are above the dynamic recrystallization temperature of the alloy ($\sim$ 700 K).

Similar calculations for Ta_2O_5 showed the flash temperature to be in the range of 750 K. In this case, the average radius of the asperity–abrasive contact area was inferred from Fig. S4 as $\sim$ 15 μm. V was taken as 5 m/s, as for Ti-6Al-4V polishing. Again the calculated flash temperatures at the asperity–abrasive contacts are high enough, $\sim 0.4T_m$, where viscous-like flow may be expected.

S2: Graph representation of topological evolution

The phenomena of bridging of the neighboring asperities is analyzed as an evolving random planar graph $G(t) = (V, E(t))$ where the nodes V represent the asperities and the edge weights $E(t)$ denote the propensity of a pair of neighboring nodes to bridge evolving over time. As observed from the *in situ* electron micrographs (see Fig. S5), the evolving morphological features, here the particle morphology, are embedded in a two-dimensional plane. To ensure that the graph-based model is consistent with the planar disposition of the surface features, we subscribe to the following planar graph representation in this study:

Definition: A graph $G = (V, E)$ with nodes V and edge weights connecting any pair of nodes denoted by E is planar if it can be drawn in a plane without edges intersecting.

In other words, every planar graph G can be embedded into a two-dimensional plane $\mathbb{R}^2$ such that nodes i and j are connected if and only if there exists an isomorphic representation where disks representing nodes i and j touch each other only at the boundary, i.e., no overlapping interiors; see Fig. S6 for examples. This formal representation allows for the description of morphological features, including asperities and undulations as circles (disks) in the plane or nodes V in the graph G. Edge weight $E(t)$ captures the evolution of the neighborhood structure $N_i(t)$ about each node i during the process (i.e., the distribution of distances $\rho_{ij}, j \in N_i(t)$ of all the immediate neighbors of i).

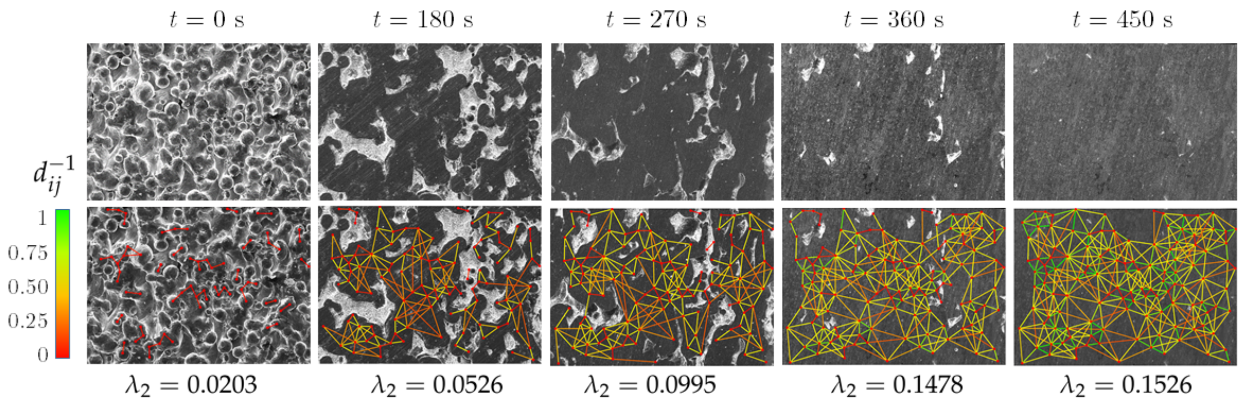

FIG. S5. Topological evolution of the Fiedler number (λ_2) as a function of time. Top row shows a series of SEM images taken at 90 s interval. The corresponding asperity network in each of these stages is shown in the bottom row.

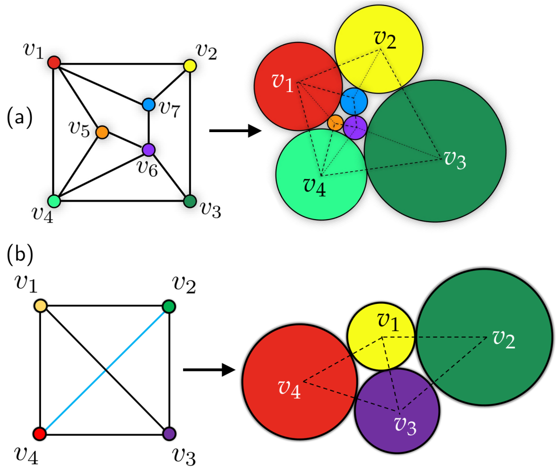

FIG. S6. Examples showing planar and non-planar graphs by embedding in a two-dimensional plane using disks. (a) Shows a planar graph G1: disks representing the nodes in graph G1 intersect at the boundary whenever the nodes are connected (b) shows a non-planar graph G2: nodes v_2 and v_4 in graph G2 after embedding do not intersect at the boundary.

Given the neighborhood structure $N_i(t)$ of node i, the propensity $e_{ij}(t) \in E(t)$ of a node i to bridge with its nth nearest node $j \in N_i(t)$ can be expressed as:

$$e_{ij}(t) \equiv P(b_{ij}(t)|N_i(t)) \propto P(b_{ij}(t)) \times P(N_i(t)|b_{ij}(t)) \tag{6}$$

Here, $P(b_{ij}(t))$ denotes the prior, i.e., the probability of bridging before the neighborhood structure, $N_i(t), \forall i \in V$ is gathered from the SEM image sequence. In the absence of information on $N_i(t)$, a uniform distribution is assigned to $P(b_{ij}(t))$ as an initial (non-informative) prior. To capture the neighborhood information $N_i(t)$ from these images, we note that the asperities on the sample surface (as estimated from SEM images, see Fig. S5) follow a homogeneous Poisson distribution. From the distribution theory, we can show that the square of inter-asperity distance $\rho^2_{ij}(t) = ||v_i - v_j||_2 - (r_i(t) + r_j(t))$ of an asperity i to its nth nearest neighboring asperity j follows a chi-square distribution with $2n$ degrees of freedom. Therefore, we have:

$$e_{ij}(t) \equiv P(b_{ij}(t)|N_i(t)) \propto \frac{\xi_{ij}^{n-1}(t)e^{-\xi_{ij}(t)/2}}{(n-1)!} \tag{7}$$

where $\xi_{ij}(t) = 2\pi\lambda\rho^2_{ij}(t)$, λ is the asperity surface density and $\rho_{ij}(t) = ||v_i - v_j||_2 - (r_i(t) + r_j(t))$.

From the *in situ* SEM image sequence shown in Fig. S5(a), we notice that as the pol-

ishing process ensues, asperities progressively bridge and is reflected by an increase in the connectivity of the representative asperity network (Fig. S5(b)). To quantify this evolution pattern (we show in the sequel that this quantification would also serve to validate the planar random graph model), we track the second smallest eigenvalue λ_2 (also called the Fiedler value) of the graph Laplacian, $L(t) \triangleq \mathcal{D}(t) - E(t)$ where $\mathcal{D}(t)$ is the diagonal matrix representing the degree of each node and is given as:

$$\mathcal{D}(t) = \left[\sum_{j=1}^{N} e_{1j}(t) \ \ldots \ \sum_{j=1}^{N} e_{Nj}(t) \right] \tag{8}$$

It has been established that the second largest eigenvalue of $L(t)$ captures the algebraic connectivity in the graph, also called the Fiedler number (λ_2) [S4, S5].

The lower bound on λ_2 is calculated using the geometric embedding of planar graph on a unit sphere as presented in [S6], where each of the nodes are represented by non-overlapping semi-spherical caps of radius $r_i, i \in V$. For the micrograph in Fig. S5, $|V| = 121$. A strongly connected network of asperities can be assumed as an ideal close packing of uniform spheres such that each node is connected to at most 6 nearest neighbors. Under such conditions it can be shown that $0.16 \leq \lambda_2 \leq 0.39$ holds. The initial value of $\lambda_2 = 0.0203$ (see Fig. S5, bottom row) indicates that the degree of each node is < 1. After 450 s of polishing, λ_2 increases to 0.1526 suggesting a minimum degree of 6 among all neighboring asperities. The network structure along with the corresponding λ_2 values is summarized in Fig. S5. Additionally, the linear increase in the value of λ_2 suggests that there are significant topological changes in the surface even during the final stages of polishing process which otherwise are not reflected in the S_a or S_v measurements (see Fig. 7 in the main text).

[S1] H. Blok, in *Proceedings of the general discussion on lubrication and lubricants*, Vol. 2 (London: IMechE, 1937) pp. 222-235.

[S2] H. S. Carslaw and J. C. Jaeger, *Conduction of Heat in Solids* (Clarendon Press, Oxford, 1959).

[S3] X. Tian and F. E. Kennedy, Journal of Tribology **116**, 167 (1994).

[S4] F. R. K. Chung, *Spectral Graph Theory*, Vol. 92 (American Mathematical Society, RI, 1997).

[S5] P. K. Rao, O. F. Beyca, Z. Kong, S. T. Bukkapatnam, K. E. Case, and R. Komanduri, IIE Transactions **47**, 1088 (2015).

[S6] D. A. Spielman and S. H. Teng, Linear Algebra and its Applications **421**, 284 (2007).
